# Successful pre-operative local control of skin exposure by sarcoma using combination of systemic chemotherapy and Mohs’ chemosurgery

**DOI:** 10.1186/s12957-020-01815-w

**Published:** 2020-02-11

**Authors:** Manabu Hoshi, Tadashi Iwai, Naoto Oebisu, Hiroaki Nakamura

**Affiliations:** Department of Orthopedic Surgery, Osaka City University Graduate School of Medicine, 1-4-3 Asahi-Machi, Abeno-ku, Osaka, 545-8585 Japan

**Keywords:** Malignant wound, Mohs’ chemosurgery, Sarcoma, Skin exposure, Surgery

## Abstract

**Background:**

Sarcomas sometimes invade the skin and become exposed, producing malignant wounds characterized by bleeding, exudate, odor, and infection. Malignant cutaneous sarcomas are generally incurable and ultimately impair patients’ quality of life. Mohs’ chemosurgery is a previously published technique for chemical fixation of a cutaneous tumor and subsequent excision.

**Case presentation:**

We present the case of a 44-year-old man with an undifferentiated pleomorphic sarcoma arising in the right chest wall and rupturing through the skin. The tumor manifested as a malignant wound with ulceration, bleeding, exudate, and a strong odor. Treatment with systemic chemotherapy and Mohs’ chemosurgery was initiated. After repeated courses, the tumor demonstrated significant shrinkage. We were then able to perform wide resection and reconstruction with a rectus abdominis musculocutaneous flap. Pathologic examination of the resected specimen confirmed negative margins.

**Conclusions:**

Mohs’ chemosurgery with concurrent systemic chemotherapy is an effective and reliable treatment option for achieving pre-operative local control of sarcomas that rupture through the skin.

## Background

Sarcomas arise from every part of the human body, and they sometimes penetrate the skin and become exposed. The resulting dermal lesion is often characterized by continuous bleeding, exudate, a strong odor, and infection.

In 1941, Frederic E. Mohs developed a technique for the chemical fixation and subsequent excision of cutaneous tumors using a paste (Mohs’ paste) containing zinc chloride; he published this method, describing it as a “chemical technique” [1, 2]. Recently, the combined effect of conventional therapy with bio-nanotechnology has become an increasingly attractive treatment option [3]. In particular, the zinc chelator contained in Mohs’ paste functions as a matrix metalloproteinase inhibitor, which contributes to the management of vascular disease [4]. In the case presented here, Mohs’ chemosurgery and concurrent systemic chemotherapy was administered, and successful local control of the cutaneous manifestation of the sarcoma was achieved.

Written informed consent was obtained from the patient prior to publication of this case report.

## Case presentation

**Fig. 1:**
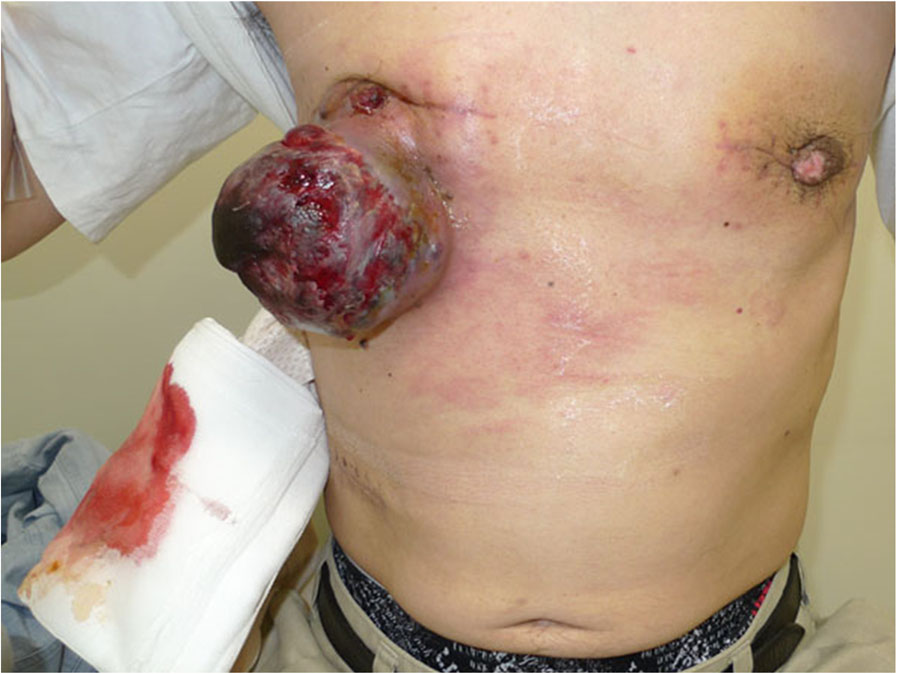
A malignant wound was associated with the tumor of the right breast. Skin ulceration, bleeding, exudate, a strong odor, and infection were observed

Two months prior to presentation at our hospital, a 44-year-old man presented at another hospital with a gradually growing tumor in his right breast. He had also noticed a tumor in the left breast 20 years prior. He underwent tumor resections in both breasts at the same time. Recurrence of the tumor in the right breast was discovered 2 weeks after the initial surgery. Due to the rapid growth of this recurrent tumor, he was referred to our hospital for treatment. Macroscopically, the tumor in the right breast measured 12.0 cm in diameter; it was exudative, exhibited ulceration and bleeding, and gave off an odor (Fig. 1).

**Fig. 2:**
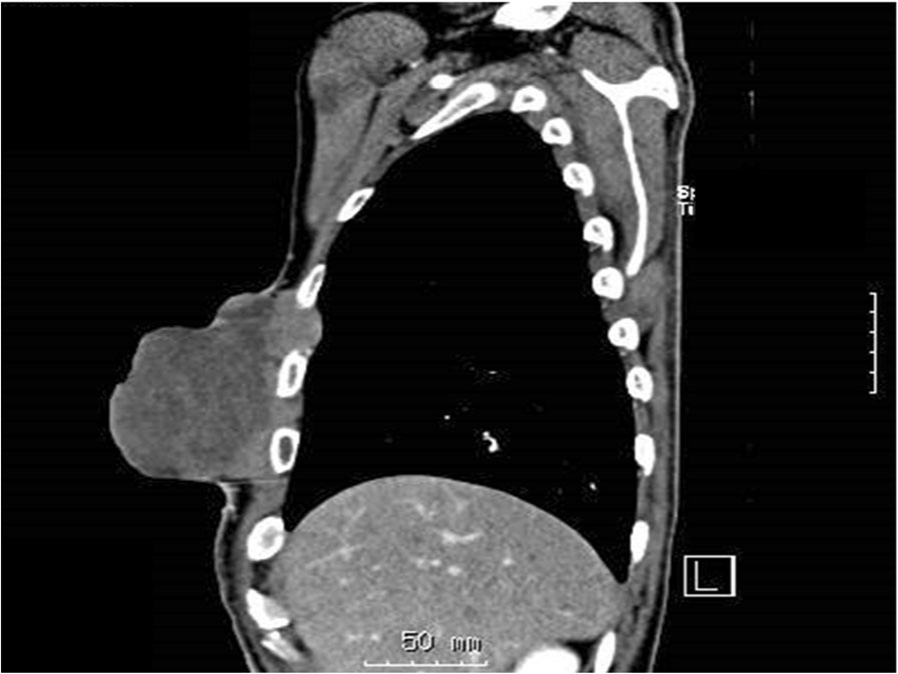
Sagittal computed tomography scan showing the tumor protruding from the chest wall. The tumor also invades the intercostal area

**Fig. 3:**
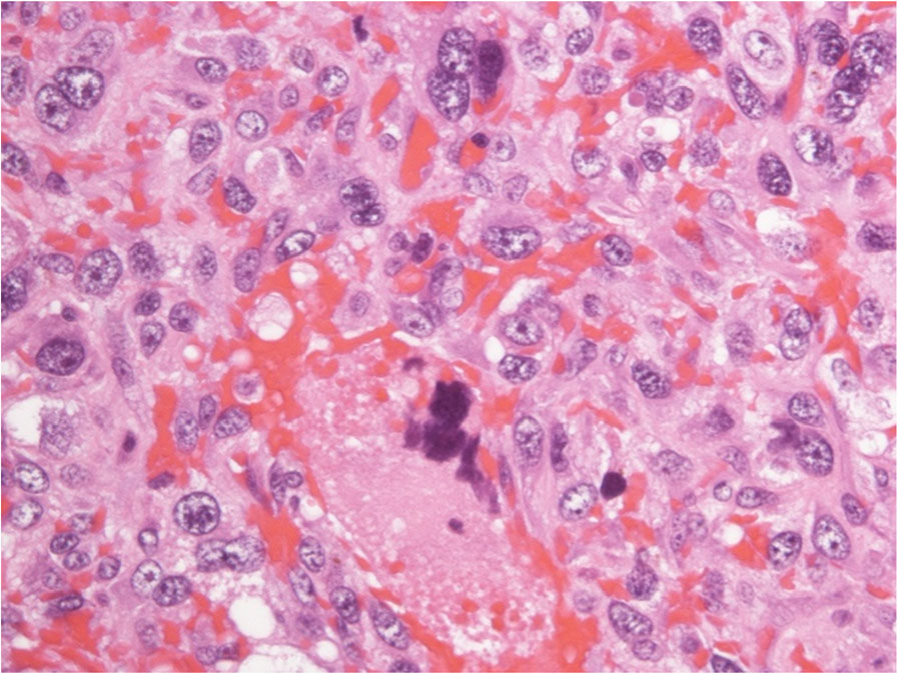
The pathological diagnosis confirmed high-grade sarcoma consistent with undifferentiated pleomorphic sarcoma (hematoxylin and eosin stain; × 400).

Computed tomography (CT) scan showed a massive mass measuring 10 cm × 7 cm × 9 cm (Fig. 2). No metastatic lesions were observed. The pathological diagnoses of the specimens resected at the previous hospital were pleomorphic sarcoma of the right breast and atheroma of the left breast, consistent with undifferentiated pleomorphic sarcoma (Fig. 3).

**Fig. 4:**
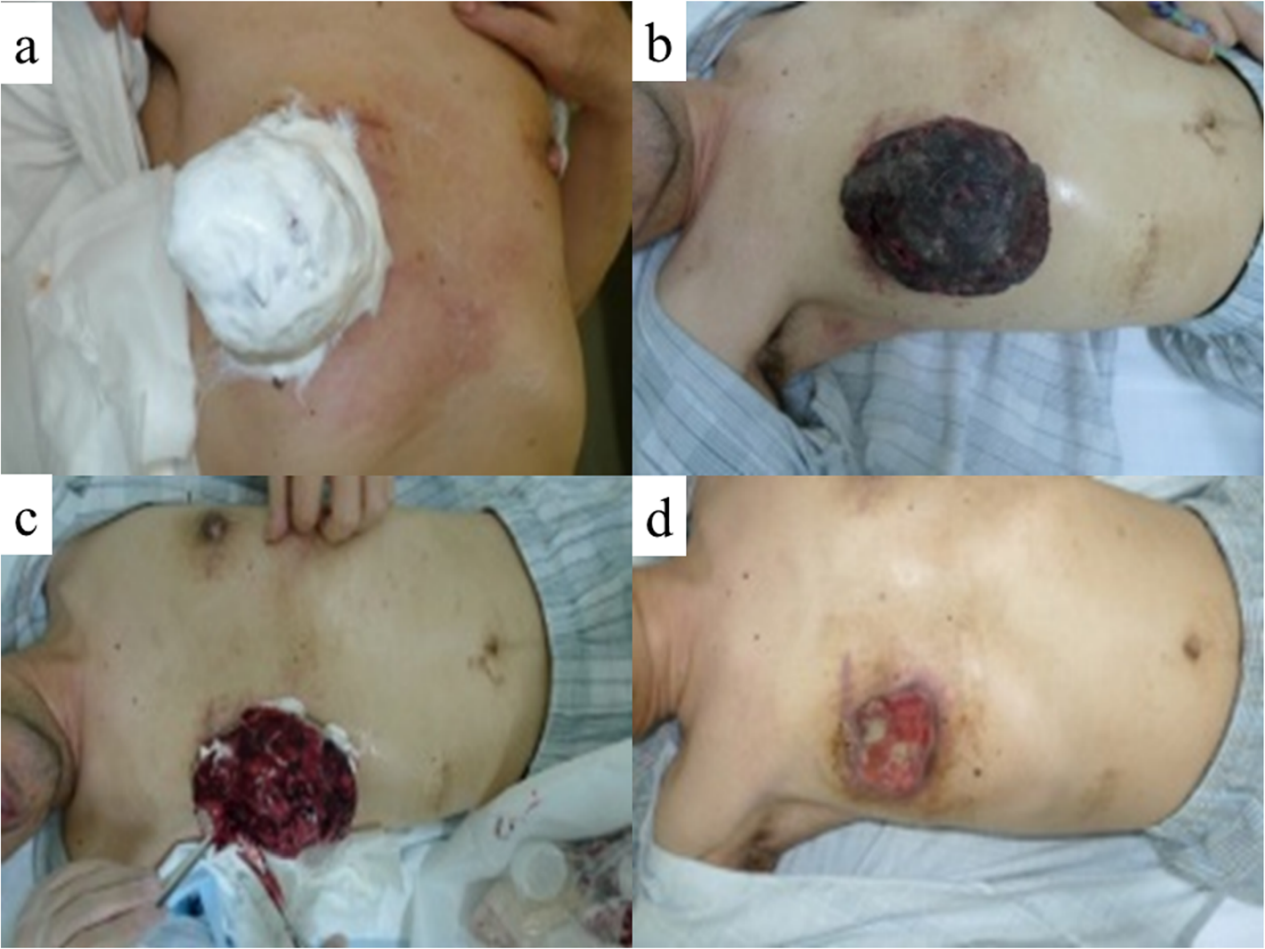
**a** The surface of the tumor has been painted with Mohs’ paste. **b** The surface of the malignant wound has been chemically fixed; it became dry, black in color, and hard. **c** Appearance after cutting the surface of the tumor. **d** Pre-operative physical appearance of the chest wall. The tumor has shrunk remarkably.

The patient was treated with combination therapy consisting of chemotherapy and Mohs’ chemosurgery. The chemotherapy regimen was performed according to the K2 protocol [5]. Prior to the application of Mohs’ paste, we applied lidocaine jelly to the normal skin surrounding the tumor because the paste can induce pain in healthy skin. We then painted petroleum jelly on the normal skin around the tumor to prevent Mohs’ paste from directly contacting the normal skin. Using wooden tongue depressors, we painted Mohs’ paste on the tumor, applying pressure to active bleeding sites (Fig. 4a). It took 10–20 min for oozing from the sarcoma to stop. The extra paste was then wiped off with saline-soaked gauze, completing the procedure. Most surface bleeding points can be controlled with this brief treatment. Following treatment, the surface of the malignant wound became dry, black in color, and hard (Fig. 4b). We next cut the degenerative surface of the tumor using surgical scissors (Fig. 4c), and we again pressed Mohs’ paste to the bleeding points of the tumor for some minutes. We repeated this procedure every 3–4 days.

**Fig. 5:**
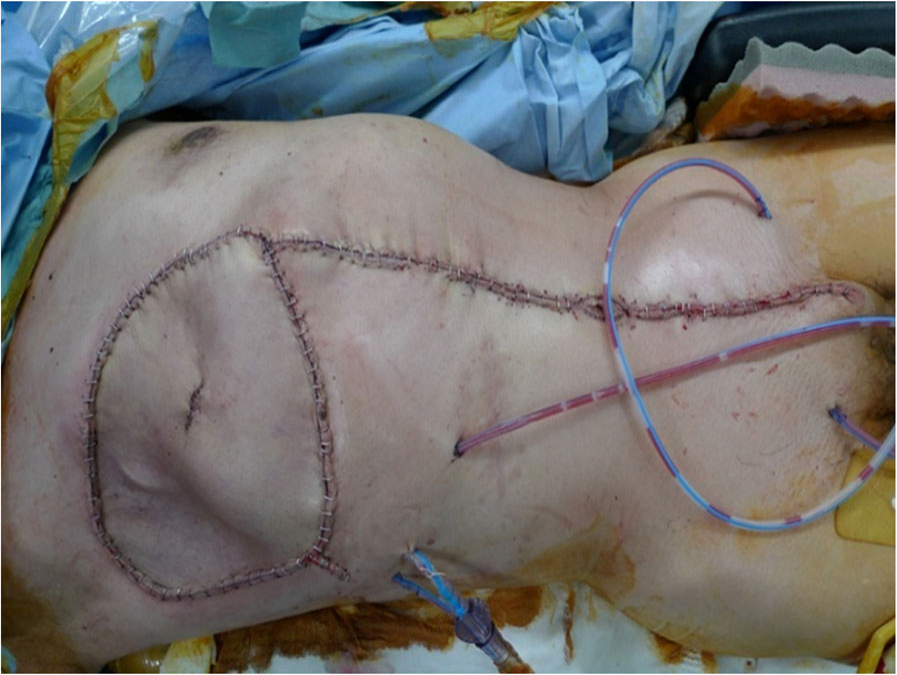
Wide resection and reconstruction with a rectus abdominis musculocutaneous flap were performed

Following three courses of chemotherapy combined with Mohs’ chemosurgery, the tumor had remarkably shrunk in size (Fig. 4d). We performed wide resection of the tumor and reconstruction with a rectus abdominis musculocutaneous flap (Fig. 5). Pathological examination of the resected specimen confirmed the presence of fibrosis and foam cells in most parts of the tumor, suggesting that the pre-operative treatments were effective, but variable tumor cells were also found in small numbers. The surgical margins were negative. The patient received three courses of chemotherapy after surgery and was discharged from the hospital. However, bilateral metastatic lesions in the lungs emerged 6 months postoperatively, and the patient died of these metastases 18 months after surgery despite additional chemotherapy.

## Discussion and conclusions

In the case presented here, of a patient presenting with skin invasion from a pleomorphic sarcoma in the chest wall, the combination of Mohs’ chemosurgery and systemic chemotherapy was an effective and safe method of pre-operative local treatment. Frederic E. Mohs originally published this technique for chemically fixing a cutaneous tumor [1, 2]. Mohs’ paste comprises a mixture of zinc chloride, distilled water, zinc powder, and glycerin. Zinc chloride has multiple beneficial features: it penetrates into the tissue well, allows precise control of fixation depth, does not interfere with subsequent second-intention healing, is not systemically toxic, is safe to handle, and lacks odor [6].

It is converted into zinc ions in the presence of exudate, and these zinc ions precipitate wound proteins. Ultimately, tissues, vessels, and the tumor are chemically fixed [7].

Mohs’ chemosurgery has primarily been used to treat patients with skin cancer [8], head and neck cancer [9], breast cancer [10], and even genital cancer [11]. To date, there is little reported clinical evidence regarding the application of Mohs’ chemosurgery for tumor reduction in sarcoma patients in the orthopedic field [12, 13]. Sarcoma tumor tissue is fragile and contains many abnormal small vessels; accordingly, sarcomas that invade the skin are often associated with bleeding. Such bleeding is detrimental to patients, and it is difficult to control. Direct suturing and electric coagulation have poor success rates in achieving hemostasis and sometimes increase bleeding further. In contrast, chemical fixation using Mohs’ paste is a safe and reliable method of accomplishing hemostasis of the tumor. As well, treatment of sarcomas located on the chest wall, as in the case presented here, present a risk for pneumothorax, and therefore procedures must be performed with caution. Thus, from a safety perspective, the method of chemical fixation using Mohs’ paste that is presented here provides a safe, feasible, and effective treatment alternative compared to previously reported methods (Table 1) [12, 13].

**Table 1:** A review of the literature concerning Mohs’ chemosurgery surgery for deep-seated soft tissue sarcoma

Author	Year	Patient age/Sex	Anatomical site	Tumor size	Histology	Chemotherapy	Follow-up	Outcome	Ref.
Chiller et al.	2004	31 M	Thigh	3.5 cm	Myxofibrosarcoma	None	14 months	No recurrence	[12]
Nemoto et al.	2019	74 M	Buttock	15 cm	Dedifferentiated liposarcoma	+	6 months	No recurrence	[13]
Present case	2020	44 M	Chest wall	12 cm	Dedifferentiated liposarcoma	+	18 months	No recurrence	

In patients with sarcomas that invade the skin, frequent dressing changes are required to cope with bleeding, exudates, odor, and/or infection [14]. Mohs’ paste is effective in reducing these symptoms, thereby contributing to improvements in patients’ quality of life. Moreover, from an economical viewpoint, frequent dressing changes with hemostatic agents are expensive. In the case presented here, the frequency of dressing changes was reduced with the use of Mohs’ paste. A reduction in medical costs can therefore be anticipated in association with this treatment.

In our patient with skin invasion from a pleomorphic sarcoma, multimodal treatment involving chemotherapy and Mohs’ chemosurgery contributed to tumor shrinkage, achieving successful pre-operative local control of the malignant wound, and enabled surgical tumor resection. Our results hint that Mohs’ chemosurgery may be an effective and reliable treatment option for sarcomas that rupture through the skin.

## Data Availability

All data obtained is available within the manuscript.
